# Technical Challenges of Real-Time Adaptive MR-Guided Radiotherapy

**DOI:** 10.3389/fonc.2021.634507

**Published:** 2021-03-08

**Authors:** Daniela Thorwarth, Daniel A. Low

**Affiliations:** ^1^Section for Biomedical Physics, Department of Radiation Oncology, University of Tübingen, Tübingen, Germany; ^2^Department of Radiation Oncology, University of California, Los Angeles, Los Angeles, CA, United States

**Keywords:** MR-linac, MR-guided radiotherapy, biologically adaptive radiotherapy, MR-only radiotherapy, online adaptive radiotherapy, real-time adaptive radiotherapy

## Abstract

In the past few years, radiotherapy (RT) has experienced a major technological innovation with the development of hybrid machines combining magnetic resonance (MR) imaging and linear accelerators. This new technology for MR-guided cancer treatment has the potential to revolutionize the field of adaptive RT due to the opportunity to provide high-resolution, real-time MR imaging before and during treatment application. However, from a technical point of view, several challenges remain which need to be tackled to ensure safe and robust real-time adaptive MR-guided RT delivery. In this manuscript, several technical challenges to MR-guided RT are discussed. Starting with magnetic field strength tradeoffs, the potential and limitations for purely MR-based RT workflows are discussed. Furthermore, the current status of real-time 3D MR imaging and its potential for real-time RT are summarized. Finally, the potential of quantitative MR imaging for future biological RT adaptation is highlighted.

## Introduction

The development of radiation therapy (RT) technology has enabled radiation oncologists to conform radiation doses to a level that was assumed to be physically impossible during the first 90 years of the field. Tumor margin prescriptions were developed to account for the differences between the radiation dose distribution and the patient’s anatomy based on patient positioning errors, anatomical changes, and intrafraction motion (1). In-room imaging went a long way to reduce the margins needed to account for positioning and anatomical changes, but intrafraction motion remained a challenge due to the lack of real-time internal imaging of soft tissues (2, 3). This limitation was solved with the recent development of magnetic resonance (MR)-guided RT, defined as the integration of a radiation-delivery machine and an MR scanner (4, 5). While intra-fraction and real-time imaging became more straightforward, the improved soft tissue contrast of MR and the relatively low level of artifacts made this modality the first practical platform for adaptive RT (6–12).

While MR imaging delivers no ionizing radiation, some acquisition protocols are limited due to tissue heating, which restricts some of the real-time imaging protocols, especially for high magnetic field systems. The clearance between the patient and the machine is also smaller than for conventional linear accelerators, limiting patient positioning strategies. The impact of the main magnetic field on the delivered radiation dose can be profound, and unlike computed tomography (CT), maintaining adequate spatial accuracy requires great care and needs to be checked routinely. Still, with all of these caveats, MR-guided RT (MRgRT) is likely to revolutionize some RT treatments, especially those that need high spatial resolution soft tissue imaging each fraction or real-time imaging for linear accelerator gating.

The aim of this review is to discuss technical challenges in the field of real-time adaptive MRgRT and to highlight current directions of research which reach out for new technical solutions to provide a basis for future clinical achievements in this field.

## Magnetic Field Strength Tradeoffs

One of the more obvious differences between the commercial MRgRT systems is their main magnetic field strengths (4, 5). Current systems span the range of 0.35 to 1.5 T, inviting the question of what, if any, are the tradeoffs between the different magnetic field strengths? Radiology’s history of MR imaging main magnetic field strengths may imply that greater magnetic field strengths always provide better images than can be acquired at lower magnetic field strengths. Because the images are produced by the net polarization of water protons, which is proportional to the main magnetic field, the number of protons available to produce the radiofrequency (RF) signal required for image acquisition increases with increasing field strength. All other things being equal, the subsequent signal to noise ratio (SNR) increases due to the increased number of polarized protons.

The purpose of MRgRT systems is to treat cancer, and since all commercial systems treat with x-rays, generally accepted clinical quality and accuracy specifications, e.g., established by the International Commission on Radiation Units and Measurements (ICRU), should be met, as should imaging accuracy. The core functionality of MRgRT systems is not to mimic diagnostic systems, and as such the benchmark for their imaging performance should not come from diagnostic radiology requirements, but from radiation oncology requirements (13, 14).

The requirements of dose distribution accuracy and image fidelity can be examined independently. With respect to dose distribution accuracy, the AAPM stipulates that the overall accuracy goal is that the delivered dose should agree with the physical dose to within 5%, a specification that includes uncertainties in machine calibration and dose calculation accuracy (15). While x-rays themselves are not impacted by the magnetic field, the secondary electrons are. When an external magnetic field is applied, the electrons are influenced by the Lorenz force, causing them to travel in a circle, but because of their many medium interactions, the overall paths are instead distorted in the direction of the Lorenz force. This distortion increases with increasing magnetic field. For larger radiation fields in a homogeneous water phantom, this distortion affects only the lateral beam penumbra. As the fields get small with respect to their secondary electron range, the entire high dose region distorts towards the Lorenz force direction (16, 17). When heterogeneities such as air cavities are encountered, the curved electron trajectories caused by the Lorentz force cause those secondary electrons to return to the exit cavity surface, substantially increasing the dose at those surfaces. These surfaces include tissue-air interfaces (such as bowel gas, the trachea, or nasal cavities) and tissue-lung interfaces, and the dose hot spots can be as large as 48% for a 1.5 T MRgRT system (18). State-of-the-art dose calculation software utilizes Monte Carlo transport calculations that model the influence of the magnetic fields, so the calculated dose can meet the accuracy requirements for a static patient, but changes in the heterogeneity distribution between the simulation scan and the patient’s anatomy during the treatment can cause the doses at these interfaces to differ substantially from the calculated doses. That said, the relative dose heterogeneities can be somewhat compensated by overlapping beams from different directions. Finally, exit skin dose can exhibit the same behavior as internal heterogeneities, causing the skin exit dose to considerably higher than it would in a non-MRgRT treatment (19, 20).

Importantly, radiation dosimetry of air-filled ionization chambers is significantly influenced by the presence of a static magnetic field. To account for this effect during absolute dosimetry, dedicated field strength and chamber type and orientation specific correction factors need to be identified *via* measurements or simulations (21–25).

The imaging fidelity can be summarized as image quality for organ delineation, and geometric accuracy. It is generally considered that MRgRT systems provide image quality that is adequate for its intended purpose. MR images are generated using magnetic field gradients and an assumption of the knowledge of the relationship between the magnetic field strengths and position. Errors in this relationship cause the imaged features to appear offset from their actual positions. CT-based IGRT geometric alignment specification tolerance is 1 mm (26). Published spatial accuracy of the commercial MRgRT systems show that the 0.35 T system meets the 1 mm specification within 5 cm radius from isocenter and a 2 mm specification at 17.5 cm from isocenter (27), while the 1.5 T system has a 1.1 mm maximum spatial distortion within 20.0 cm from isocenter (28, 29).

Machine-based magnetic field errors are not the only source of MR image distortion. The patient chemical makeup will also modify the local magnetic field and therefore the apparent position of an imaged structure. Such susceptibility artifacts or chemical shifts lead to shifts in the imaged positions of anatomical structures which are proportional to the magnetic field. For human tissues, these can be in the order of millimeters for 1.5 T scanners (30), while the same artifact at 0.35 T would be much lower. For specific sequences, the susceptibility artifacts can be reduced by increasing the RF bandwidth, which has the corresponding side-effect of reducing SNR, reducing, but not eliminating the advantage of the increased field strength.

Finally, the radiofrequency energy emitted by the MR scanner is absorbed by the human body, heating the body. The term used to describe this for clinical MR scans is the specific absorption rate (SAR). The amount of heating is proportional to the square of the magnetic field strength (31), so limiting the SAR will be more challenging for the higher field MRgRT systems. Limiting the SAR may be most challenging when conducting real-time imaging for purposes of linear accelerator gating. Two of the “selling points” of MR are that it does not deliver ionizing radiation and that it can provide real-time internal imaging, so restricting this imaging due to SAR concerns would reduce the perceived benefit of MRgRT.

## Real-Time MR Auto-Segmentation

Current clinical experience of online adaptive MRgRT shows that one of the main bottle necks is the lack of fast and accurate segmentation of MR images. As the requirement for real-time adaptive MRgRT is to robustly provide MR-based structure delineations in the order of seconds, deep learning (DL) approaches have been investigated recently by several groups (32–36). Most DL models for auto-contouring were trained so far for the pelvic region providing to generate organ structures based on MR images (32, 34). Additionally, DL concepts for contour propagation from simulation images to daily MR have been proposed recently (37). Since adaptive MRgRT is a novel clinical application, annotated MR data for model training and validation is sparse, thus alternative approaches such as cross-modality learning have been explored (38). Even though numerous challenges remain concerning real-time MR-based auto-segmentation, first investigations regarding the clinical implementation have reported fast (few seconds) and robust use in MRgRT of prostate cancer (39).

## MR-Only Planning

During real-time adaptive MRgRT, treatment planning as well as dose calculation need to be conducted for every RT fraction, but a CT simulation is no longer available. Consequently, approaches for MR-based dose planning—so called MR-only planning workflows—have been proposed to support real-time adaptive MRgRT.

MR-only planning in combination with MR-simulation for RT planning without the need of additional planning CT has been previously proposed (40, 41). Early on, mechanistic models using dedicated MR sequences, such as e.g., Dixon-based sequences, were proposed to generate synthetic CT data sets based on MR imaging data (42). Alternative approaches proposed voxel-intensity based approaches to translate MR signal values into synthetic CT readings (42). Dosimetric studies analyzing the accuracy of radiation dose calculation using synthetic CT reported dose differences on the order of 0.5% relative to CT-based dose simulation (43). Today, several commercial products for synthetic CT generation are available and studies reporting first clinical experience using MR-only simulation for RT planning have been recently published (44).

Because the acquisition of dedicated MR sequences for synthetic CT determination is time consuming, online adaptive MRgRT in today’s clinical practice mostly relies on extremely simplified methods to generate synthetic CT information, such as bulk density assignments to anatomical structures. This simplification may compromise the dosimetric accuracy as currently robust conversion approaches from MR to CT are lacking. To overcome this, several groups have recently proposed deep learning models for the calculation of synthetic CT data sets based on anatomical MR imaging which has shown to be a time-efficient and robust approach (45–48). Dosimetric evaluations have shown promising results in terms of dose differences of 0–0.5% (49, 50). However, online quality assurance of synthetic CT seems to be challenging and bears dosimetric risks especially when it comes to the anatomical location and electron density assignment of bony structures. The use of undistorted MR images for synthetic CT generation is crucial in the light of real-time high precision MR-guidance. Nevertheless, the use of artificial intelligence (AI) tools for MR-only workflows may open new opportunities for real-time adaptive MRgRT using hybrid MR-linacs.

## Real-Time MRI

One of the most compelling features of MRgRT is the ability to conduct real-time imaging (51, 52). Real-time imaging provides unparalleled visualization of internal organs to enable the clinicians to monitor and ultimately limit the impact of intra-fraction motion. This motion may be due to peristalsis, bladder filling, or breathing. The MRgRT system will provide a sequence of images, typically at a few Hertz. This sequence is typically started immediately after any setup images are acquired where the tumor is identified, and the patient moved to account for relative shifts or deformations. If the motion is due to breathing, the sequence is typically visualized for a few breathing cycles to determine the amount of motion. If gating is available and desired, a gating window is defined by segmenting the target or a suitable surrogate and applying a margin to act as a gating structure. The MRgRT system then tracks that gating structure for each image frame and monitors whether the structure is within the gating window, typically to within a pre-selected percentage. Note that this process is currently 2D, due to a lack of commercially available real-time 3D imaging sequences. Recent studies however showed promising approaches towards real-time 3D MR image acquisition (53–56) and reconstruction (57).

A critical concern is the latency between the time, the images are acquired and the time the machine or operator can respond to an undesired motion. This is related to the amount of time required to acquire the image data, to reconstruct the data, and to analyze the data and determine that a significant deviation has occurred. The latter could be the time required for the system to conduct the contouring, the determination that the motion should trigger a change in machine state and implement that change (beam on or off) or the time a human operator would take to evaluate and manually change the machine state. The latency of the image acquisition step is typically assumed to be approximately half of the image acquisition time because the image is expected to reflect the average state during the acquisition time. The remaining latency sources are functions of the hardware and software that the manufacturer employs. These times should be short, especially for free breathing motion gating, where multiple gating events will take place during a treatment.

Recent studies have shown latency times of real-time 3D MR imaging on MR-linac systems of 300–500 ms (58). Quality assurance to verify real-time interventions should be performed using a dynamic phantom that contains MR-imageable structures and the ability to do both point (ion chamber) and area (film) dosimetry. An end-to-end test of a gated treatment that uses clinically realistic treatment times and gating windows will determine if the latency significantly degrades the treatment dose accuracy (59).

## Online Adaptive RT

With the advent of 4D-MR imaging with minimal latency times, real-time adaptive RT seems to be one of the next technological steps of MR-guided radiotherapy. Consequently, it might be possible to irradiate moving targets with highest precision using MLC-tracking based on real-time MR readings. MLC-tracking based on CBCT imaging has been proposed earlier and proven to be suitable for clinical usage (60). A major challenge of real-time adaptive RT is the methodology of real-time dose calculation or reconstruction. Fast et al. (61) proposed a tool for online dose reconstruction which determined the delivered dose based on pre-calculated dose influence data in less than 10 ms. After initial investigations of online dose reconstruction based on 2D cine MR images (62) and 3D cine MR in addition to treatment log files (63), recent studies proposed deep learning strategies to empower real-time dose calculation and motion prediction (64, 65). Even though proposed for offline planning, methods for deep learning-based dose prediction seem to be promising tools to support real-time dose reconstruction (66, 67). In the light of current trends for reduced number of RT fractions, dose adaptation and calculation based on real-time anatomical information gets more and more important.

Accurate dose assessment of fractionated RT requires deformable dose accumulation for targets and OARs. So far, no clinically usable solution has been proposed for this problem. Therefore, robust algorithms for 4D dose accumulation are required to provide precise voxel-readings of recorded, locally varying dose distributions for better TCP and NTCP estimation (68).

Nevertheless, clinical real-time adaptive MRgRT needs thorough quality assurance and testing. To date, dedicated end-to-end tests were proposed to specifically test certain aspects of adaptive MR-guided RT (7, 59, 69–71). Future end-to-end test developments may focus on ways to evaluate real-time imaging, dose calculation, and accumulation. Furthermore, mechanisms that ensure robust and safe radiation delivery need to be implemented in real-time MR guided workflows.

## Functional Imaging

In addition to the enormous potential of MRgRT for geometrical precision and adaptation in real-time, functional MR data has been shown by several studies to be prognostic for outcome after RT in different tumor entities (72, 73). Consequently, interventions steered by functional MR imaging biomarkers seem to be one of the most promising concepts towards personalized, biologically individualized RT. Even though prognostic information using functional MR imaging may also be gathered with state-of-the-art diagnostic MR systems which do not suffer from hardware limitations such as the hybrid MR-linac scanners (74), biological real-time adaptation such as image biomarker guided dose painting to overcome local tumor radiation resistances can only be realized on hybrid MR-linacs (75). Biological RT individualization in terms of dose adaptation based on imaging information requires the measurement of quantitative imaging biomarkers (76). Recent studies have shown that quantitative MR imaging is possible using hybrid MR-linacs (77). Diffusion-weighted imaging (DWI), for example, can be implemented such that robust quantitative diffusion data can be measured with high repeatability and reproducibility (77). A major challenge for using quantitative biomarkers in observational and also interventional multi-center MR-linac studies will be the validation of imaging protocols for reproducibility of quantitative imaging in order to prove that quantitative imaging biomarkers are comparable between centers (78). Furthermore, test-retest studies to assess the level of repeatability will be prerequisites for future quantitative imaging studies in different tumor entities. So far, most studies have focused on quantitative imaging assessments and on investigating prognostic value of DWI (72, 74, 77, 79–81). A further challenge will be the realization of functional interventions. Currently, echo planar MR imaging (EPI) techniques are mostly used for DWI even though these are known to be susceptible for geometrical distortions (82). However, dose painting based on functional MR data requires geometrical accuracy. Consequently, current research strategies in this field include investigation of alternative MR imaging techniques, e.g., turbo spin echo (TSE) based sequences such as SPLICE (83) or strategies to correct for geometrical distortions (82). Nevertheless, hybrid MR-linacs are a major technological innovation towards real-time biological adaptation of RT aiming for increasing tumor control rates in different cancer types in the future.

## Discussion

MR-guided RT offers high-resolution real time MR imaging before and during RT and allows thus to adapt for inter- and intra-fraction changes. Consequently, smaller target margins and potentially better organ-at-risk sparing may be possible with MRgRT, opening new horizons towards single or few fraction RT delivery (84). For real-time MR-guidance, many involved steps require automatization. Researchers in different sub-fields have started to automatize and speed-up processes using AI methods. However, to generate robust and intelligent models which can assist with treatment decisions, large sets of curated, standardized and well documented data are needed for model training and validation.

Furthermore, there is evidence, that biological characteristics of the tumor microenvironment play an important role in terms of radiation resistance. Consequently, quantitative MR imaging biomarkers need to be identified as predictive for RT outcome, validated in phantom and clinical studies and might then in the future qualify for interventional, quantitative MR based RT studies.

Ultimately, all technical solutions developed to overcome challenges related to real-time adaptive MR-guided RT deserve intensive clinical validation before unsupervised usage in routine MRgRT.

## Author Contributions

DT and DL equally contributed to writing and proof-reading of this manuscript. All authors contributed to the article and approved the submitted version.

## Funding

The Tübingen MR-Linac program received funding from the German Research Council (DFG, grants no. ZI 736/2-1, TH 1528/6-1). We acknowledge support by Open Access Publishing Fund of University of Tübingen.

## Conflict of Interest

DT declares institutional collaborations including financial and non-financial support with Elekta, Philips, Siemens, PTW Freiburg, and IBA Dosimetry. DL declares institutional financial support by Varian.
